# Antioxidants Prevent the Effects of Physical Exercise on Visual Cortical Plasticity

**DOI:** 10.3390/cells12010048

**Published:** 2022-12-22

**Authors:** Gabriele Sansevero, Alan Consorti, Irene Di Marco, Eva Terzibasi Tozzini, Alessandro Cellerino, Alessandro Sale

**Affiliations:** 1 Neuroscience Institute, National Research Council (CNR), 56124 Pisa, Italy; 2 NEUROFARBA, University of Florence, 50139 Florence, Italy; 3 Stazione Zoologica Anton Dohrn, 80121 Napoli, Italy; 4 BIO@SNS Lab, Scuola Normale Superiore, 56126 Pisa, Italy; 5 Leibniz Institute on Aging-Fritz Lipmann Institute (FLI), 07745 Jena, Germany

**Keywords:** physical activity, visual cortex plasticity, mithormesis, oxidative stress, mitochondrial biogenesis, IGF-1, antioxidants

## Abstract

Background: Physical activity has been recently shown to enhance adult visual cortical plasticity, both in human subjects and animal models. While physical activity activates mitochondrial oxidative metabolism leading to a transient production of reactive oxygen species, it remains unknown whether this process is involved in the plasticizing effects elicited at the visual cortical level. Results: Here, we investigated whether counteracting oxidative stress through a dietary intervention with antioxidants (vitamins E and C) interferes with the impact of physical exercise on visual cortex plasticity in adult rats. Antioxidant supplementation beyond the closure of the critical period blocked ocular dominance plasticity in response to eye deprivation induced by physical activity in adult rats. Conclusions: Antioxidants exerted their action through a mithormetic effect that involved dampening of oxidative stress and insulin-like growth factor 1 (IGF-1) signaling in the brain.

## 1. Introduction

Physical exercise represents a non-pharmacological intervention that consistently improves general health by activating mitochondrial metabolism in skeletal muscles, and improving insulin sensitivity [1,2]. As a result of activation of mitochondrial oxidative metabolism during aerobic exercise, physical activity leads to a transient production of reactive oxygen species (ROS) [3,4]. Chronic excessive oxidative stress with ROS overproduction is implicated in various pathological conditions, such as atherosclerosis, diabetes, cancer, and neurodegeneration [5,6]. On the contrary, physiological peaks of ROS have a positive impact on the organism, qualifying this phenomenon as an example of mitohormesis, a process whereby exposure to low doses of mitochondrial stressors promotes adaptive changes that are useful for toleration of subsequent similar stress [7,8]. Accordingly, it has been shown that ROS are needed for the health-promoting effects elicited by physical activity, with diet supplementation with antioxidants precluding the beneficial impact of exercise on insulin sensitivity and mitochondrial biogenesis in young men [8,9]. These vitamins can counteract the enhanced mitochondrial biogenesis promoted by physical exercise, preventing ROS generation and leading to the inhibition of the beneficial mitohormetic response [10,11,12,13].

In parallel to its influence on metabolism, physical activity elicits a plethora of beneficial effects at the brain level, potentiating memory abilities, delaying age-related cognitive decline, increasing long-term synaptic plasticity, and enhancing levels of molecules critically involved in neuronal plasticity, such as neurotrophins, the vascular endothelial growth factor (VEGF), and the insulin-like growth factor 1 (IGF-1) [14,15,16,17]. Recent work has started to investigate the impact of physical activity on the paradigmatic model of visual cortex plasticity. Classic experiments by Wiesel and Hubel [18] first demonstrated that depriving one eye of vision [monocular deprivation (MD)] in young mammals causes a reduction in the cortical response elicited by stimulation of the deprived eye, a process called ocular dominance (OD) plasticity [19], when the treatment is applied during a critical period (CP) in the early postnatal life [18,19,20,21,22,23,24,25,26]. Recent works challenged the dogma of a strict CP, identifying strategies able to enhance plasticity in adulthood [27,28]. Remarkably, physical activity in adult subjects restores plasticity in rodent models [29,30,31,32], an effect that has been linked to multiple central actions, including sustained neurotrophin expression and activation of specific GABAergic neural circuits [17,33,34,35]. In humans, physical activity has been shown to favor recovery of visual functions and to promote homeostatic plasticity in response to short-term eye occlusion [36,37], even if replication of this latter effect has been controversial [38]. 

While information concerning the role of myokines in mediating the systemic effects of physical activity is increasing [39], the role of redox balance in cerebral plasticity remains mostly unanswered. A high metabolic demand during the critical period was reported to affect the maturation of fast-spiking interneurons in mice, inducing a significant loss of extracellular matrix perineuronal nets [40]. On the other hand, the activity of the NADPH oxidase complex, responsible for the production of ROS, is known to be crucial for the induction of long-term synaptic plasticity in the hippocampus and in the primary visual cortex of the mouse [41,42]. Despite this evidence, a direct link between mitohormesis and visual cortex plasticity is missing. Here, we focused on the paradigmatic model of OD plasticity in response to visual deprivation in one eye and evaluated, for the first time, whether modulation of oxidative stress obtained through a dietary intervention with antioxidants interferes with the plasticizing effects of physical exercise on the visual cortex of adult rats. To prevent mitohormesis, we used a diet supplementation with vitamins C and E, two antioxidants widely employed in both human and animal model research [9,10,12,43].

## 2. Materials and Methods

### 2.1. Animal Rearing

All experiments were conducted on Long-Evans black hooded rats of both sexes (males:females in a 1:1 proportion). All methods were carried out in accordance with the approved guidelines and regulations of Italian Ministry of Public Health. All experimental protocols were approved by the Italian Ministry of Public Health (approved protocol n. 144/2017-PR). Animals of both genders were housed in a room with a temperature of 22 °C and a 12 h light/dark cycle. In all experiments, experimenters were blind to experimental conditions. After weaning, animals remained in standard conditions (two to three per cage, plexiglass 40 × 30 × 20 cm), until P60-P70, receiving food (standard pellets) and water at libitum. Rats were then singled-housed and divided into four different experimental groups: running-vitamin (RV), running-control (RC), sedentary-vitamin (SV), and sedentary-control (SC). Naïve rats (without deprivation and not subjected to running) fed with either a standard diet or vitamin supplementation were also used as controls. Running animals had free access to a wheel to perform voluntary physical exercise. Vitamin animals received pellets enriched with 1000 mg/Kg vitamin E and 1049 mg/Kg vitamin C. The entire length of diet supplementation was three weeks.

For the analysis of visual cortical plasticity in young rats, P21 animals were divided in two experimental group: young SC and young SV.

### 2.2. Experimental Design and Statistical Analyses

We estimated the sample size needed for each experiment performing a power analysis by using a dedicated software (GPower). We fixed the alpha at 0.05 and beta at 0.20. The estimate of the expected difference between the experimental groups was based on the knowledge of values of the studied parameter in the control condition deriving from previous data obtained in our laboratory or, if not possible, on values in the literature. The numbers resulting from this analysis were increased by 15% in order to take into account the drop-out factor. Statistical analysis was done using the SigmaPlot Software. Data were tested for normality and equal variance before running statistical tests; parametric tests were run on normally distributed data and, when case normality test failed, non-parametric tests were performed. Differences between two independent groups were assessed with a two-tailed *t*-test. Two-way ANOVA and two-way RM ANOVA, followed by a Holm–Sidak multiple comparison procedure, were used to compare normally distributed data differing for two independent factors. Two-way RM ANOVA on ranks was used to compare not normally distributed data differing for two independent factors. Level of significance was *p* < 0.05, unless otherwise specified. The size of biological replicates is indicated by the n numbers in the various experimental sections, and also in Table 1. In the figures, means are shown and error bars represent standard error of the mean (SEM).

### 2.3. Surgical Procedures

#### 2.3.1. Adult animals

Rats were anesthetized with isoflurane. Monocular deprivation (MD) was performed through eyelid suturing during the last one of three weeks of differential training. The animals were allowed to recover from anesthesia and were returned to their cages. Eyelid closure was inspected daily until complete cicatrized. Rats showing occasional lid reopening (observed under a surgical microscope) were not included in the experiments.

#### 2.3.2. Young animals

Young SC and SV rats were fed with either control diet or vitamin supplementation, respectively, starting at P21 for a total of three weeks. During the last week, MD was performed as described for adult animals. The animals were then allowed to recover from anesthesia and were returned to their cages. Eyelid closure was inspected daily until complete cicatrized. Rats showing occasional lid reopening (observed under a surgical microscope) were not included in the experiments.

### 2.4. In Vivo Electrophysiology

After three weeks of differential training (last week with MD), the animals were anesthetized with intraperitoneal injection of urethane (1.4 g/kg; 20% solution in saline; Sigma-Aldrich) and placed in a stereotaxic frame. Body temperature was continuously monitored and maintained at ~37 °C by a thermostated electric blanket during the experiment. A hole was drilled in the skull, corresponding to the binocular portion of the primary visual cortex (binocular area V1) contralateral to the long-term-deprived eye. After exposure of the brain surface, the dura was removed, and a micropipette (2 MΩ) filled with NaCl (3 M) was inserted into the cortex 5 mm from (intersection between sagittal and lambdoid sutures). Both eyes were fixed and kept open by means of adjustable metal rings surrounding the external portion of the eye bulb. After eyelid reopening, 45 min were left before the beginning of electrophysiological recordings, and rats were maintained with both eyes open and hydrated in front of a blank stimulus. We measured ocular dominance (OD) by calculating the contralateral to ipsilateral visual evoked potential (VEP) ratio (C/I VEP ratio), i.e., the ratio of VEP amplitudes recorded by stimulating the eye contralateral and ipsilateral, respectively, to the visual cortex where the recording is performed. Recordings were performed only from the visual cortex contralateral to the deprived eye. During recording through one eye, the other was occluded by an automatic black shutter. In order to prevent side-effects due to adaption, the order of stimulation of the two eyes was randomly alternated. In each animal, VEPs were acquired using the responses recorded at 100 μm of depth first, and then at 400 μm. The C/I VEP ratios obtained at the two depths did not differ between each other; thus, they have been averaged together (paired *t*-test, *p* = 0.29 in RV rats; *p* = 0.23 in RC rats; *p* = 0.98 in SV rats; *p* = 0.44 in SC rats). Signals were band-pass-filtered (0.1–100 Hz), amplified, and fed to a computer for analysis. At each depth, at least 50 events were averaged in synchrony with the stimulus contrast reversal. Transient VEPs in response to abrupt contrast reversal (0.5 Hz) were evaluated in the time domain by measuring the peak-to-baseline amplitude and peak latency of the major negative component. Visual stimuli were horizontal sinusoidal gratings of different spatial frequencies and contrast, generated by a VSG2/2 card running custom software and presented on a monitor (20 × 22 cm; luminance 15 cd m^−2^) positioned 20 cm from the rat’s eyes. At the end of the recording sessions, the animals were euthanized with urethane overdose.

### 2.5. Western Blotting

Separate groups of rats not involved in electrophysiology were used for Western blotting. Western blot was used to quantify phosphorylated rIG1-1 protein levels in the visual cortex of rats from the four experimental groups. In order to avoid circadian effects, all animals were sacrificed (chloral hydrate (100 mg/kg) + guillotine) during the same time interval each day (10:00–12:00 h; light phase). After decapitation, brains were rapidly removed, and the visual cortex was dissected and frozen on dry ice. Proteins were extracted with lysis buffer (20 mM Tris-HCl pH 7.45, 150 mM NaCl, 10 mM EDTA, 0.1 mM Na3VO4, 1 mM PMSF, 1 μg/mL leupeptin, 1 μg/mL aprotinin, 1% Triton X-100 and 10% glycerol), and the total concentration of the samples was assessed with a protein assay kit (Bio-Rad, Hercules, CA, USA) using a bovine serum albumin-based standard curve. Protein extracts (50 μg for phosphorylated rIGF-1) were separated by electrophoresis and blotted; filters were blocked and incubated overnight at 4 °C with primary antibodies (anti phosphor-rIGF-1, 1:200, Merck-Millipore, Burlington, MA, USA). Filters were also probed with anti-α-tubulin antibody (1:15,000 dilution, Abcam, Cambridge, UK) as an internal standard for protein quantification. Blots were then rinsed in Tween buffered saline (TPBS), incubated in infrared labeled secondary antibodies IRDye 700 CW or 800 CW (1:20,000 dilution, Li-Cor Biosciences, Lincoln, NE, USA), washed in TPBS, and briefly rinsed in PBS. Filters were scanned using an Odyssey^®^ IR scanner and densitometry analysis was performed with Odyssey^®^ imaging software 3.1. Antibody signal was calculated as the integrated intensity of the region defined around the band of interest. The protein amount was evaluated measuring the signal of the band of interest and dividing it by the signal of β-tubulin band on the same filter. The results were divided by the average value obtained in each experiment for the control group (SC animals). Samples were run in duplicate.

### 2.6. Determination of IGF-1 and β-Hydroxybutyrate Concentration in Serum 

We used the same animals used for Western blotting analysis. Blood samples were collected animals between 10 and 12 AM. Rats were anaesthetized with chloral hydrate (100 mg/kg) and blood was taken through cardiac puncture. Serum is the liquid fraction of whole blood that is collected after the blood is allowed to clot (2 h, RT). The clot was removed by centrifugation (4000 rpm, 10 min) and the resulting supernatant was carefully removed. Serum IGF-1 and β-hydroxybutyrate concentrations were measured using Rat IGF-1 ELISA kit, RayBio and Beta-Hydroxybutyrate Assay Kit, Sigma-Aldricht, respectively. Both the procedures were performed following manufacturers’ guidelines. 

### 2.7. Sample Preparation for Proteome Analysis

Separate groups of rats, not involved in any other experiment, were used for proteomics. The animals were anaesthetized with isofluorane (3%) and decapitated with a guillotine. Binocular visual cortices were collected and snap-frozen in liquid nitrogen. On preparation for MS, protein amount was estimated based on fresh tissue weight (assuming 5% of protein w/w) and lysis buffer (4% SDS, 100 mM HEPES, pH 8, 1 mM EDTA, 100 mM DTT) was added accordingly to a final concentration of 1 μg/μL. Samples were then vortexed (5 times) prior to sonication (Bioruptor Plus, Diagenode) for 10 cycles (30 s ON/60 s OFF) at high setting, at 4 °C. The samples were then centrifuged at 3000× *g* for 5 min at room temperature, and the supernatant transferred to 2-mL Eppendorf tubes. Reduction (15 min, 45 °C) was followed by alkylation with 20 mM iodoacetamide (IAA) for 30 min at room temperature in the dark. Protein amounts were confirmed, following an SDS–PAGE gel of 4% of each sample against an in-house cell lysate of known quantity. A total of 100 μg protein of each sample was taken along for digestion. Proteins were precipitated overnight at 20 °C after the addition of a 4× volume (400 μL) of ice-cold acetone. The following day, the samples were centrifuged at 20,800× *g* for 30 min at 4 °C and the supernatant was carefully removed. Pellets were washed twice with 500 μL ice-cold 80% (*v*/*v*) acetone in water then centrifuged at 20,800× *g* at 4 °C. They were then allowed to air-dry before addition of 50 μL of digestion buffer (3M Urea, 100 mM HEPES, pH 8). Samples were resuspended with sonication (as above), LysC (Wako) was added at 1:100 (*w*/*w*) enzyme:protein and digestion proceeded for 4 h at 37 °C with shaking (1000 rpm for 1 h, then 650 rpm). Samples were then diluted 1:1 with Milli-Q water and trypsin (Promega) added at the same enzyme to protein ratio. Samples were further digested overnight at 37 °C with shaking (650 rpm). The following day, digests were acidified by the addition of TFA to a final concentration of 2% (*v*/*v*) and then desalted with Waters Oasis^®^ HLB μElution Plate 30 μm (Waters Corporation, Milford, MA, USA) in the presence of a slow vacuum. In this process, the columns were conditioned with 3 × 100 μL solvent B (80% (*v*/*v*) acetonitrile; 0.05% (*v*/*v*) formic acid) and equilibrated with 3 × 100 μL solvent A (0.05% (*v*/*v*) formic acid in Milli-Q water). The samples were loaded, washed three times with 100 μL solvent A, and then eluted into 0.2-mL PCR tubes with 50 μL solvent B. The eluates were dried down with the speed vacuum centrifuge and dissolved at a concentration of 1 μg/μL in reconstitution buffer (5% (*v*/*v*) acetonitrile, 0.1% (*v*/*v*) formic acid in Milli-Q water). For data-independent analysis (DIA), peptides were spiked with retention time iRT kit (Biognosys AG, Schlieren, Switzerland) prior to analysis by LC-MS/MS.

### 2.8. Data-Independent Acquisition 

Peptides were separated using the nanoAcquity UPLC system (Waters) with a trapping (nanoAcquity Symmetry C18, 5 μm, 180 μm × 20 mm) and an analytical column (nanoAcquity BEH C18, 1.7 μm, 75 μm × 250 mm). The outlet of the column was coupled to a QEHFX (Thermo Fisher Scientific, Waltham, MA, USA) using the Proxeon nanospray source. Solvent A was water, 0.1% FA, and solvent B was acetonitrile, 0.1% FA. Samples were loaded at constant flow of solvent A at 5 μL/min onto the trap for 6 min. Peptides were eluted via the analytical column at 0.3 μL/min and introduced via a Pico-Tip Emitter 360 μm OD × 20 μm ID; 10 μm tip (New Objective, Littleton, MA, USA). A spray voltage of 2.2 kV was used. During the elution step, the percentage of solvent B increased in a non-linear fashion from 0% to 40% in 120 min. The capillary temperature was set at 300 °C. The RF lens was set to 40%. Data from a subset of samples were acquired in data-dependent acquisition (DDA) in order to create a spectral library. MS conditions were as follows: full-scan spectra (350–1650 m/z) were acquired in profile mode in the Orbitrap with resolution of 60,000. The fill time was set to 50 ms with limitation of 2 × 105 ions. The “TopN = 15” method was employed to take the precursor ions (with an intensity threshold of 5 × 104) from the full-scan MS for fragmentation (using HCD collision energy, 30%) and quadrupole isolation (1.4 Da window) and measurement in the Orbitrap (resolution 15,000, fixed first mass 120 m/z), with a cycle time of 3 s. MS/MS data were acquired in profile mode (QEHFX). Only multiply charged precursor ions were selected. Dynamic exclusion was employed (15 s and relative mass window of 10 ppm). Isotopes were excluded. For data acquisition and processing of the raw data, Xcalibur 4.0 (Thermo Scientific, Waltham, MA, USA) and Tune version 2.9 were employed. For the data independent acquisition (DIA), the same gradient conditions were applied to the LC as for the DDA and the MS conditions were varied as described: full-scan MS spectra with mass range 350–1650 m/z were acquired in profile mode in the Orbitrap with resolution of 120,000. The filling time was set at maximum of 20 ms with limitation of 5 × 105 ions. DIA scans were acquired with 34 mass window segments of differing widths across the MS1 mass range with a cycle time of 3 s. HCD fragmentation (30% NCE) was applied, and MS/MS spectra were acquired in the Orbitrap with a resolution of 30,000 over the mass range 200–2000 m/z after accumulation of 2 × 105 ions or after filling time of 70 ms (whichever occurred first). Ions were injected for all available parallelizable time. Data were acquired in profile mode.

### 2.9. DIA Data Analysis 

For library creation, the DDA and DIA data were searched using Pulsar in Spectronaut Professional+ (version 12.0.20491.0.21234, Biognosys AG, Schlieren, Switzerland). The data were searched against a species-specific (Mus musculus Swiss-Prot) database and a list of common contaminants. The data were searched with the following modifications: carbamidomethyl (C) (Fixed) and oxidation (M)/acetyl (protein N-term; variable). A maximum of two missed cleavages for trypsin were allowed. The identifications were filtered to satisfy FDR of 1% on peptide and protein level. The generated library contained 74,254 precursors, corresponding to 4729 protein groups. Relative quantification was performed in Spectronaut for each pairwise comparison using the replicate samples from each condition. Precursor matching, protein inference, and quantification were performed in Spectronaut using default settings. The data (candidate table, Table S1) were then exported, and further data analyses and visualization were performed with R-studio using in-house pipelines and scripts.

For differential analysis, the ion intensity of the peptide spectral matching (PSM) was log2 transformed, normalized, and summarized into protein group quantities by taking the median value. At least two unique peptides per protein were required for the identification. Differential protein expression was assessed using the limma package (DOI: 10.1093/nar/gkv007). Differences in protein abundances were statistically determined using Student’s *t*-test moderated by the empirical Bayes method. *p* values were adjusted for multiple testing using the Benjamini–Hochberg method (FDR, denoted as “adj. P”) (Benjamini & Hochberg, 1995). The results are reported in Dataset EV2.

## 3. Results

### 3.1. Physical Activity in Adulthood Reopens the CP for OD Plasticity in the Visual Cortex

**Figure 1:**
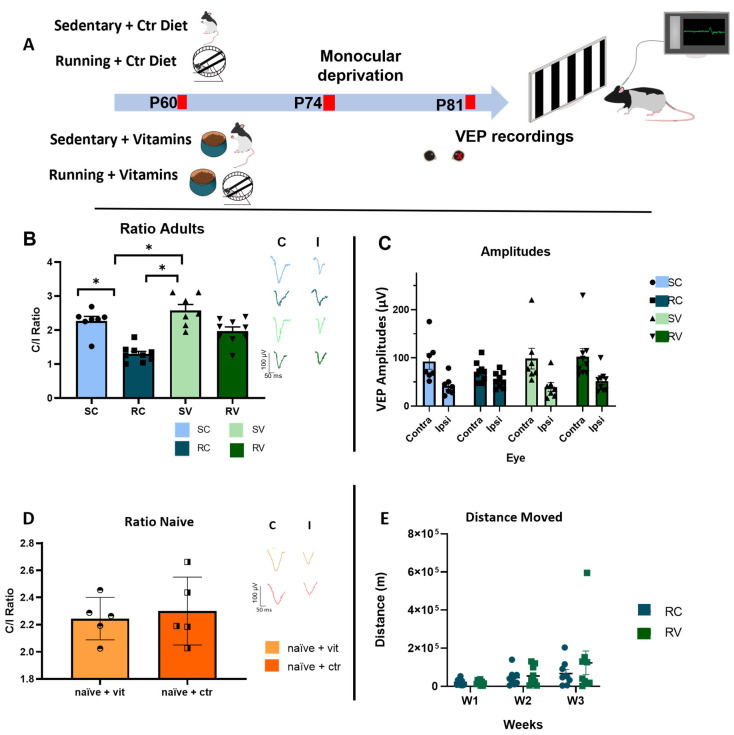
Antioxidant supplementation dampens running-induced visual cortical plasticity in adult rats. (**A**) Schematic diagram of the protocol. (**B**) Ocular dominance (OD) was determined by measuring the controlateral to ipsilateral (C/I) ratio in the amplitude of VEPs. In adult animals, running induced a marked OD shift in rats fed with the control diet (RC rats, n = 9) in response to MD, with a C/I VEP ratio of 1.30 ± 0.08. The C/I VEP ratio in RC rats was significantly lower than that of sedentary rats fed with the standard diet (SC rats, n = 7, C/I VEP ratio = 2.27 ± 0.13,) (two-way ANOVA, F = 38.63, Holm–Sidak method, *p* < 0.001). Animals treated with antioxidants (RV, n = 9) displayed a significant blockade of the OD shift elicited by MD, in comparison with RC rats (RV, C/I VEP ratio = 1.97 ± 0.12 vs. RC, *p* < 0.001). Vit E and C did not per se affect OD plasticity in sedentary animals subjected to MD (SV, C/I VEP ratio = 2.58, n = 7, SV vs. SC, *p* = 0.115) Moreover, treatment with Vit E and C did not affect the baseline C/I VEP ratio in non-deprived naïve rats subjected to antioxidant supplementation, in which the C/I VEP ratio did not differ from that of naïve rats treated with control diet (naïve + vit, C/I VEP ratio = 2.24 ± 0.07; naïve + control diet, C/I Vep Ratio 2.30 ± 0.11; *p* = 0.792). (**C**) VEP amplitudes of both the contralateral and ipsilateral eye, in the different experimental groups (**D**) VEP ratio of nNaïve + vitamins (NV) and Nnaïve + Control diet (NC). (**E**) Distance moved by RC and RV animals; no difference was observed between the two groups (two-way RM ANOVA, *p* = 0.666, F = 0.196). * indicates statistically significant differences; error bars represent SEM.

In order to investigate the impact of voluntary physical activity on adult visual cortex plasticity, we compared OD plasticity in response to 7d MD in two groups of adult rats: (i) exercised animals, provided for three weeks with the possibility of completely free running (in cages endowed with a running wheel) and fed with a standard dietary regimen (running-control diet rats, RC) and (ii) sedentary animals reared in conventional standard conditions and fed with the same diet (sedentary-control diet rats, SC). A group of naïve (non-deprived) animals was used as control (naïve + ctr rats). The experimental protocol is depicted in Figure 1A. 

To determine OD, we measured the contralateral to ipsilateral (C/I) ratio in the amplitude of visual evoked potentials (VEPs) from the binocular portion of the primary visual cortex in response to a low spatial frequency grating (0.1 c/deg). The C/I VEP ratio is in the 2.0–3.0 range for adult normal rats, reflecting the predominance of crossed fibers in retinal projections (see Sale et al., 2007 and Figure 1D, naïve + ctr animals). RC rats (n = 9) showed a marked OD shift in response to MD, with a C/I VEP ratio of 1.30 ± 0.08 (Figure 1B) (Contralateral eye, C = 70.79 ± 6.8 µV; Ipsilateral eye, I = 54.24 ± 5.3 µV, Figure 1C). The C/I VEP ratio in RC rats was significantly lower than that of SC rats (C/I VEP ratio = 2.27 ± 0.13, n = 7, Figure 1B; C = 92.46 ± 16.03 µV; I = 39.56 ± 9.36 µV, Figure 1C)), and it was also significantly lower than that of naïve + ctr animals (n = 5, C/I VEP ratio 2.29 ± 0.11, Figure 1D; Contralateral eye, C = 70.79100.17 ± 76.8 µV; Ipsilateral eye, I = 54.2445.79 ± 5.328 µV) (two-way ANOVA, F = 25.65, Holm–Sidak method, *p*  <  0.001). Thus, exposure to three weeks of voluntary physical exercise restored OD plasticity in adult animals beyond the end of the CP.

### 3.2. Antioxidants Block Adult Plasticity Induced by Physical Exercise, but Not Juvenile Plasticity during the CP

Since it has been shown that the beneficial effects induced by physical activity on glucose metabolism and mitochondrial biogenesis are blocked by antioxidant supplementation [9], we asked whether counteracting oxidative stress could also have an impact on the physical exercise capability to enhance plasticity in the adult visual cortex. To answer this question, separate groups of either running (RV), sedentary (SV), or naïve (naïve + ctr) were fed with a diet supplemented with the antioxidants vitamin E and vitamin C (see Methods for details). To control for vitamin assumption, we quantified, in the two groups of animals, the daily consumption of food pellets for the entire duration of the three-week physical training period, obtaining an accurate estimate of the number of vitamins consumed by each individual, based on the known vitamin concentration in the food. The amounts of vitamin E and vitamin C did not differ between RV and SV rats, ruling out any possible side-effects due to differences in the dosage between running and sedentary animals (RV, Vit E = 27.8 ± 1.85 mg/day, Vit C = 26.45 ± 1.75 mg/day; SV, Vit E = 28.68 ± 1.67 mg/day, Vit C = 27.71 ± 1.32 mg/day, n = 9 for RV and n = 7 for SV; two-way ANOVA, F = 0.0105, *p* = 0.919). 

Strikingly, vitamin supplementation markedly reduced the plasticizing effects of physical exercise in adult rats, with treated animals (n = 9) displaying a significantly blockade of the OD shift elicited by MD, in comparison with RC rats (n = 9) (RV, C/I VEP ratio = 1.97 ± 0.12, Figure 1B; C = 102.23 ± 17.01 µV; I = 51.72 ± 7.29 µV, Figure 1C vs. RC; two-way ANOVA, F = 38.63, Holm–Sidak method, *p* < 0.001). Vitamins E and C did not per se affect OD plasticity in sedentary animals subjected to MD (SV, C/I VEP ratio = 2.58 ± 0.17, Figure 1B; C = 98.41 ± 21.43 µV, I = 39.56 ± 9.36 µV, Figure 1C, n = 7, SV vs. SC two-way ANOVA, F = 25.65, Holm–Sidak method, *p* = 0.115). Moreover, treatment with Vitamin E and C did not affect the C/I VEP ratio in naïve + vit rats, in which the C/I VEP ratio did not differ from naïve + ctrl animals treated with the control diet (naïve + vit, C/I VEP ratio = 2.24 ± 0.07; naïve + control diet, C/I Vep Ratio 2.30 ± 0.11; two-way ANOVA, F = 25.65, Holm–Sidak method, *p* = 0.792, Figure 1D). 

Importantly, we found that treatment with antioxidants did not affect the amount of voluntary physical activity, as measured in the animals used for electrophysiological recordings. RC and RV rats, indeed, did not differ from each other in terms of meters/week run across the training days (RC, n = 9; RV, n = 9; two-way RM ANOVA on ranks, F = 0.19649, Holm–Sidak method, time × diet *p* = 0.66662; Figure 1D,E). Moreover, the variability of the amount of running did not differ between RC and RV animals (equal variance test: *p* = 0.733). These results rule out the possibility that supplementation with antioxidants might reduce the beneficial effects of physical exercise on visual cortex plasticity by directly affecting motor activity levels. 

**Figure 2:**
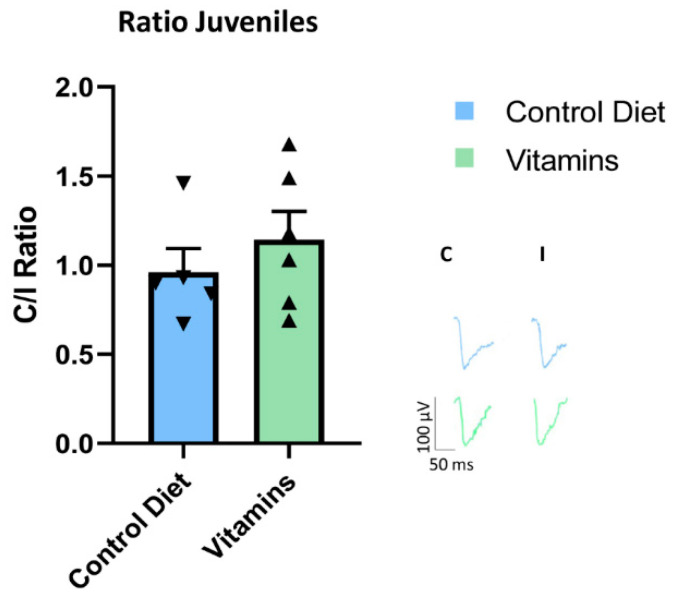
Vitamin treatment did not block plasticity during the critical period. Young rats that received vitamins C and E supplementation (SV, n = 6) did not show any difference of OD plasticity in comparison with young rats fed with the standard diet (SC, n = 5) (*t*-test; SV, ratio = 1.14 ± 0.15 vs. SC, ratio= 0.96 ± 0.13, *p* = 0.41). Error bars represent SEM.

To rule out the possibility that vitamins E and C dampen OD plasticity via a direct action on visual cortical circuits, we further studied the effects of antioxidant supplementation in a group of young animals at the peak of their CP for OD plasticity (see Methods). Vitamin supplementation for a period equal to that used in adult rats did not block OD plasticity in young rats subjected to 7d MD (Figure 2). In these animals, indeed, the C/I VEP ratio displayed a reduction in response to MD that was not statistically different from that of age-matched controls fed with the standard diet (*t*-test; SV, n = 6, ratio = 1.14 ± 0.15 vs. SC, n = 5, ratio= 0.96 ± 0.13, *p* = 0.41). Thus, antioxidants did not hinder visual cortex plasticity per se, but exerted a specific effect of blocking at the level of the enhancement of adult visual cortex plasticity induced by physical exercise. 

### 3.3. IGF-1 as a Mediator of the Effects of Physical Exercise on the Visual Cortex

The exact mechanisms whereby physical exercise signals to the brain to promote neural plasticity are not well understood. Here, we focused on two possible molecular candidates for this effect, i.e., β-hydroxybutyrate and IGF-1. 

**Figure 3:**
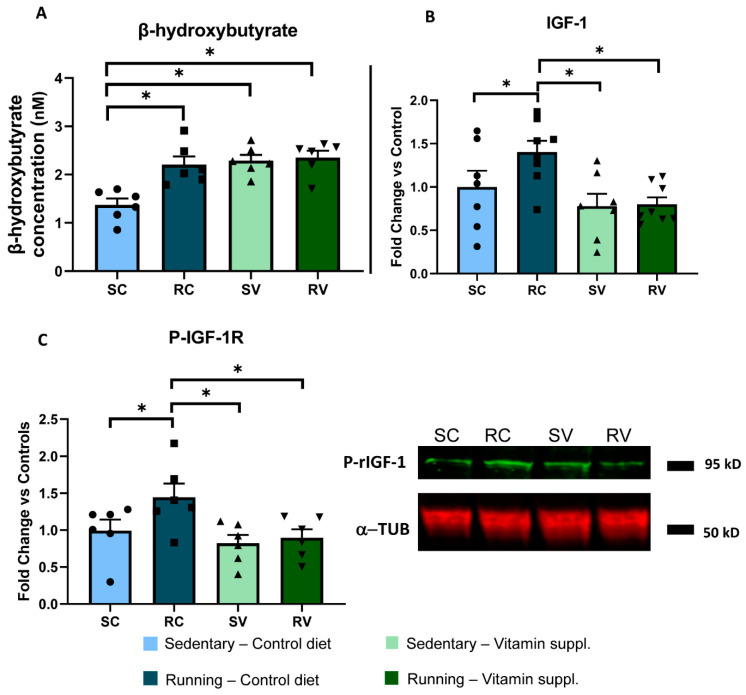
Vitamin supplementation blocks IGF1 increase in running animals. (**A**) Colorimetric assay for serum β-hydroxybutyrate. Running induced an increase in circulating β-hydroxybutyrate with respect to the sedentary condition (RC, n = 6; 2.20 ± 0.17 nM; vs. SC, n = 6; 1.37 ± 0.13 nM; two-way ANOVA, F = 7.17, Holm–Sidak method, *p* < 0.001). Vitamin supplementation caused an increase of β-hydroxybutyrate levels in sedentary animals (SV, n = 6; 2.29 ± 0.12 nM; *p* < 0.001), but no further increase was observed after free running. (**B**) ELISA for serum IGF-1. Serum IGF-1 levels were markedly increased in exercised rats as compared to sedentary animals fed with the control diet (n = 8 and n = 7, respectively; two-way ANOVA, F = 1.92, Holm–Sidak method, *p* = 0.047). Vitamin supplementation completely abolished the IGF-1 increase induced by physical exercise, without any effect in sedentary animals (n = 8 and n = 7, *p* = 0.003 and *p* = 0.006, respectively). (**C**) Western blot for pIGF-1 receptor in the visual cortex. Physical activity led to a marked increase of phosphorylated rIG1-1 in the visual cortex of RC (n = 6) vs. SC (n = 6) animals; vitamin supplementation completely blocked this increase, with RV rats (n = 6) displaying levels of phosphorylated IGF-1 significantly lower than those displayed by RC rats (two-way ANOVA, F = 1.74, Holm–Sidak method, *p* = 0.037, *p* = 0.013, respectively). No effect was found in the visual cortex of sedentary animals fed with the diet supplemented with antioxidants (n = 6) (SC vs. SV, *p* = 0.407). The right panel shows representative bands for all groups. To calculate the fold change for both (**B**,**C**) panels, each individual value was normalized, dividing it by the mean value of the SC control group. * indicates statistically significant differences; error bars represent SEM.

β-hydroxybutyrate is a ketone body released by peripheral muscles after prolonged physical exercise and is responsible for the activation of *Bdnf* gene expression in the brain, through a specific effect mediated by inhibition of HDAC activity [44]. We found that running induced an increase in the concentration of circulating β-hydroxybutyrate with respect to the sedentary condition (RC, n = 6; 2.20 ± 0.17 nM; vs. SC, n = 6; 1.37 ± 0.13 nM; two-way ANOVA, F = 7.17, Holm–Sidak method, *p* < 0.001). Vitamin supplementation induced an increase of β-hydroxybutyrate levels in sedentary animals (SV, n = 6; 2.29 ± 0.12 nM; *p* < 0.001), but no further increase was observed after free running (Figure 3A). These results strongly suggest that the enhancement of visual cortex plasticity elicited by physical exercise was not mediated by β-hydroxybutyrate. 

Then, we focused on IGF-1, a trophic factor mostly produced and released by the liver in response to physical exercise under the action of the growth hormone [45,46] and able to cross the blood brain barrier and to promote plasticity in cerebral circuitries [47,48]. We found that serum IGF-1 levels were markedly increased in exercised rats as compared to sedentary animals fed with the control diet (n = 8 and n = 7, respectively; two-way ANOVA, F = 1.92, Holm–Sidak method, *p* = 0.047) (Figure 3B). Importantly, vitamin supplementation completely abolished the IGF-1 increase induced by physical exercise, without any effect in sedentary animals (n = 8 and n = 7, *p* = 0.003 and *p* = 0.006, respectively). Thus, IGF-1 appears as a likely mediator of the enhancement of visual cortex plasticity induced by physical exercise.

To validate this hypothesis, we assessed IGF-1 signaling directly in the visual cortex, the very site of plasticity in our model. First, we measured levels of the phosphorylated active form of IGF-1 receptor (rIGF-1) in the visual cortex. We found that while physical activity led to a marked increase of phosphorylated rIG1-1 in the visual cortex of RC (n = 6) vs. SC (n = 6) animals, vitamin supplementation completely blocked this increase, with RV rats (n = 6) displaying levels of phosphorylated IGF-1 significantly lower than those displayed by RC rats (two-way ANOVA, F = 1.74, Holm–Sidak method, *p* = 0.037, *p* = 0.013, respectively). No effect was found in the visual cortex of sedentary animals fed with the diet supplemented with antioxidants (n = 6) (SC vs. SV, *p* = 0.407) (Figure 3C).

### 3.4. Global Analysis of the Effects of Physical Activity and Antioxidants on Protein Expression

In order to obtain an unbiased global view on the possible mechanisms by which physical activity enables brain plasticity and to confirm the mitigation of this effect by antioxidant supplementation, we performed mass-spectrometry based proteomics from samples of the visual cortex. The following four conditions were analyzed: standard cage (SC), standard cage + vit E and C (SV), wheel running in standard cage (RC), and wheel running + vit E and C (RV). All analyses were performed in animals that were allowed binocular vision, in order to investigate possible changes in the protein milieu induced by different environmental conditions before the induction of MD. Indeed, we reasoned that these changes in protein expression might reflect the probability for the visual cortex to successively display OD plasticity, once MD is applied. A label-free approach with data-independent acquisition (DIA) was undertaken and an average of 3520 protein groups were quantified in each sample.

**Figure 4:**
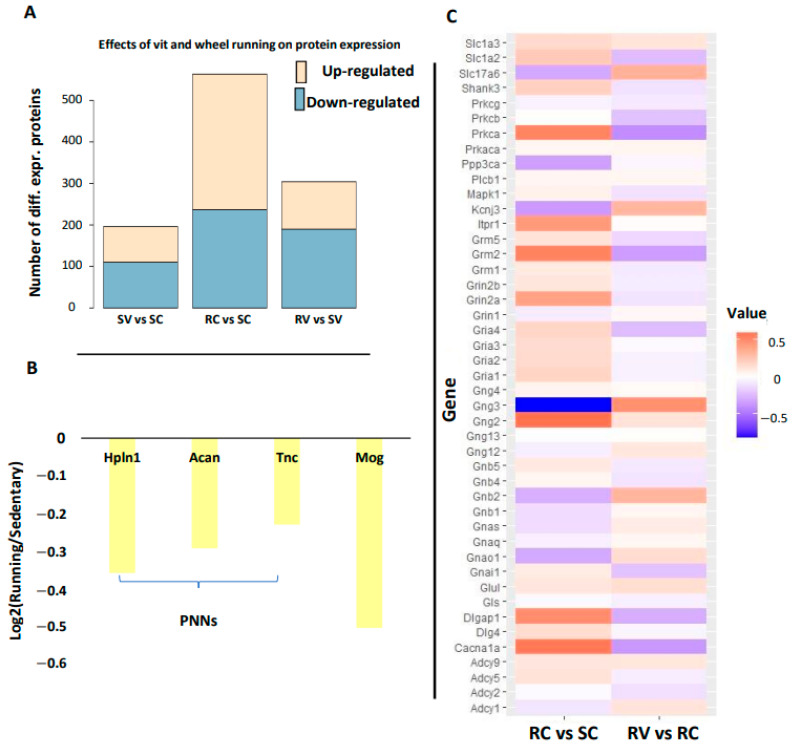
Mass-spectrometry based analysis of protein expression from visual cortical samples. (**A**) The number of differentially expressed proteins in RC vs. SC and RV vs. SV contrasts. Pink bars indicates up-regulation and grey bars down-regulation. The fraction of up-regulated proteins over the total number of detected proteins is significantly decreased in the RV vs. SV contrast (11.1 % vs. 3.9%, *p* < 10^−16^, Fisher’s exact test). (**B**) Regulation of key plasticity brakes in RC vs. SC. The average fold change is reported. Tnc = tenascin, Acan = aggrecan and Halpn1 = Hyaluronan and proteoglycan link protein 1 are components of the perineuronal nets (PNNs). Mog = Myelin oligodendrocyte glycoprotein is a constituent of myelin. All differences are statistically significant. (**C**) Regulation of proteins belonging to the KEEG Pathway “Glutamatergic synapse” rno04724 in the RC vs. SC and in the RV vs. RC comparisons displayed as heatmap. Out of 114 genes in the pathway, 45 were detected at the protein level. Red: increased expression; blue: decreased expression. Vitamin supplementation counteracts the up-regulation of protein involved in the glutamatergic signaling.

As shown in Figure 4A, in the contrast SV vs. SC (q-value < 0.01, abs(log2 (fold-change) > 0.2) 86 proteins were up-regulated and 110 were down-regulated; in RC vs. SC (Figure 3A) (q-value < 0.01, abs(log2(fold-change) > 0.2), 327 proteins were up-regulated and 236 were down-regulated, with a significant prevalence of up-regulation (*p* = 0.008, Fisher’s exact test). On the other hand, the contrast RV vs. SV resulted in 115 up-regulated and 190 down-regulated proteins, with a significant reduction in the fraction of up-regulated proteins over total detected proteins with respect to RC vs. SC. (11.1% vs. 3.9%, *p* < 10^−16^, Fisher’s exact test). Since the up-regulated proteins were highly related to plasticity (see below), this result, together with those observed at the electrophysiological level, supports the hypothesis that vitamins E and C blunt the plasticizing effects of wheel running. 

Down-regulated differentially expressed proteins (DEPs) in RC vs. SC rats included plasticity-related proteins with negative action on plasticity, such as Tnc, Acan, and Hapln1, which are components of the perineuronal nets [49], key structures enwrapping synapses on GABAergic cells that exerts a negative control on plasticity, and myelin oligodendrocyte protein (Mog), which likewise exerts a negative regulation of synaptic plasticity [50] (Figure 4B). On the other hand, proteins belonging to the pathway “glutamatergic synapse” (KEGG rno04724) were up-regulated in the RC vs. SC and RV vs. RC conditions, in line with many results indicating that physical activity promotes synaptogenesis. Strikingly, the up-regulation of this pathway in the RC condition was strongly reduced in the RV condition [14,15,16] (Figure 4C). 

**Figure 5:**
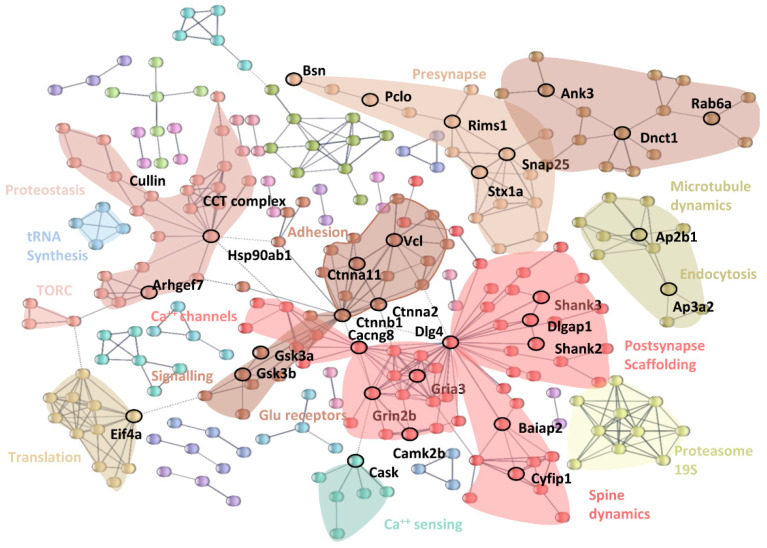
STRING Interactome of the up-regulated proteins in the contrast RC vs. SC. Each node in the graph represents a protein. Edges represent experimentally proven physical interactions with high confidence. Singletons, i.e., up-regulated proteins whose interactors are not up-regulated, are not displayed. Colors identify different clusters generated by Markov Cluster Algorithm (MCL) with parameter 1.3. Clusters particularly relevant for plasticity are highlighted as convex hulls of a different color and the names of particularly relevant genes/hubs are also displayed.

To obtain a compact representation of the differentially expressed proteins (DEPs) and to investigate systematically plasticity-related proteins, we used STRING [51], a database of protein interaction, to visualize the interactome of DEPs. In this visualization, the edges connecting protein represent experimentally validated interactions (Figure 5). The signature that we observed for up-regulated DEPs in RC vs. SC animals was consistent with a global induction of proteins related to glutamatergic, but not GABAergic transmission. Accordingly, the largest proportion of entries in the interactome of up-regulated DEPs corresponds to proteins in the excitatory postsynaptic compartment and this clearly indicates that wheel-running is associated with an increase in the excitatory drive. This module has a hub in Dlg4 (PSD95), a major constituent of the postsynaptic density that acts as a bridge between Glutamate receptors, Calcium/calmodulin-dependent protein kinase (CamK) and scaffolding proteins [52], and therefore, represents a key player in coordinating the density of glutamate receptors and in orchestrating the downstream plasticity events triggered by activation of CamK [53]. The module also contains a submodule of dendritic Ca^++^ channels and a module containing proteins that regulate the spine cytoskeleton, and regulators of local translation, such as Cyfip1. A second related module has, as hub, β-catenin (Ctnnb1), and is subdivided in a module related to junctions containing, for example, Vinculin (Vcl). 

The postsynaptic module is directly connected, via CamK2b, to a module that has, as hub, the CASK kinase, known to interact with CamKII to regulate neuronal growth and plasticity [54].

The network of postsynaptic and plasticity-controlling proteins was mirrored by a cluster of increased levels in presynaptic proteins, including Snap25, Rims1, Basson, Piccolo, and Syntaxins.

A number of other protein modules appear to be directly connected to plasticity mechanisms. These modules include: (i) protein synthesis: plasticity requires protein synthesis [55] and it has been known since the 1970s that enriched environment increases protein synthesis in the visual cortex [56]; increased protein synthesis is reflected as an increase in tRNA synthesis, eukaryotic initiation factors and, most importantly, components of the TOR complex; (ii) proteostasis, up-regulation of a module with Hsp90ab1 as hub, which is also involved in the trafficking of AMPA receptors [57]; (iii) 19S Proteasome, which plays a role in plasticity, dendritic spine formation, and regulation of postsynaptic glutamate receptors [58]; (iv) the clathrin/adaptor protein complex 2 (AP-2), which mediates the retrieval of a subset of proteins of the synaptic vescicles, including glutamate and GABA transporters [59]; and (v) proteins essential for microtubule dynamics, such as Ankryns (with Ank3 as hub), a protein necessary for synaptic maintenance [60], which is responsible for the trafficking of neurotrophins [61], and Rab6a, which is associated to mobile presynaptic vescicles and cargos [62], including neurotrophin bound to receptors [63]. The increase in protein related to neurotrophin trafficking is likely related to the well-known increase in BDNF production in the visual cortex induced by physical exercise [64,65].

Analysis of down-regulated DEPs did not reveal any module of synaptic proteins and no strongly connected modules, except for an enrichment of ribosomal proteins (rno03010, FDR < 0.001, GSEA). 

**Figure 6:**
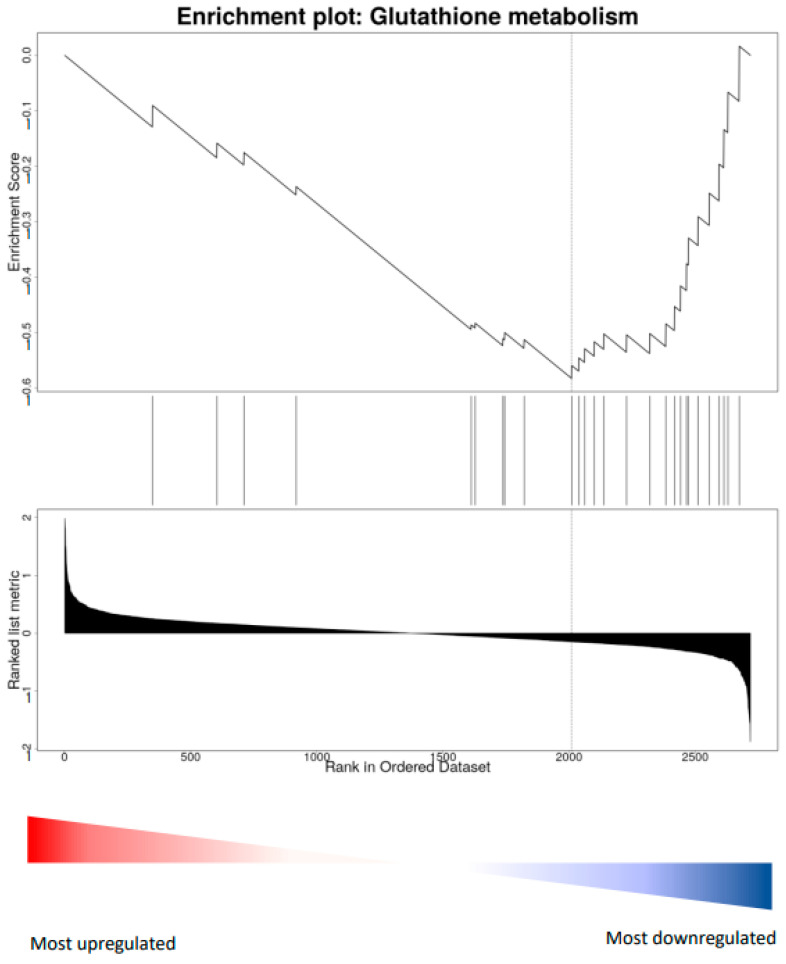
Example of relevant gene set enrichment analysis. Wheel-running vs. sedentary. Illustrations were generated in WEBGESTALT, all measured proteins were ranked based on fold change. The position in the ranked list of proteins belonging to rno00480 glutathione metabolism is shown as a vertical line. The plot of the enrichment score illustrates the enrichment of these proteins on the right (down-regulated) side of the list.

In parallel to the action on peripheral IGF-1, physical activity might promote visual cortex plasticity acting directly on the visual cortex through a regulation of oxidative stress in cortical cells. To investigate this possibility, we focused on possible signs of a local production of ROS induced by physical exercise at the visual cortical level, as is the case in the muscle. Wheel running, however, induced a significant down-regulation of Catalase and Sod1, as well as of the Glutathione metabolism pathway (KEGG, rno00480, FDR = 0.02, GSEA, NES = −1.83) (Figure 6). Thus, since running did not induce a local increase of oxidative stress in the visual cortex, the effects of vitamin treatment could not be explained as deriving from the local dampening of oxidative phosphorylation pathways.

## 4. Discussion

Physical activity is known to enhance neuronal plasticity, acting both systemically and at the local brain circuitry level. At least two mechanisms were proposed to mediate this effect: on the one hand, molecular mediators (called myokines) can be released by the muscle in the bloodstream, and influence the brain via systemic effects [45,66]. On the other hand, local cortical plasticity is critically regulated by modulation of the excitatory/inhibitory balance set in motion by physical activity, as in the case of the visual system [34,67,68], with running modulating a regulatory circuit, due to the activation of the midbrain locomotor region, that directly enhances plasticity of visual cortical neurons via modulation of the inhibitory circuits, independently of actual muscle engagement and induction of aerobic metabolism [69]. 

In addition, aerobic exercise induces a spike in ROS and previous studies have found that this spike is necessary to induce the peripheral metabolic effects of physical exercise, a phenomenon named mitohormesis [8]; accordingly, administration of antioxidants prevents the peripheral effects of exercise [9]. 

In the present study, we tested whether exercise-dependent oxidative stress is necessary to promote visual cortex plasticity in adult rats. Three weeks of voluntary physical activity performed in adults beyond the closure of the CP promoted a marked OD shift in response to MD, a plasticizing effect that parallels what previously reported for adult amblyopic rats exposed to environmental enrichment strategies [29,31,70]. Environmental enrichment and free running are associated to an increase in circulating IGF-1 that mediates, at least in part, their effects on the central nervous system [48,71]. Moreover, exogenous administration of IGF-1 in the adult visual cortex has been shown to restore the susceptibility of cortical neurons to MD [72]. Here, we found that the induction of visual cortex plasticity by physical exercise was paralleled by increased levels of circulating IGF-1, enhanced phosphorylation of the IGF-1 receptor in the primary visual cortex, and activation of the TOR complex. IGF-1 may increase cortical plasticity either acting directly at the neuronal level [72,73,74,75], but also exerting an indirect action on astrocytes, which are known to contribute to the regulation of OD plasticity [76] and express an IGF-1 receptor [77].

Remarkably, the global proteomic signature in running animals was entirely consistent with the induction of a complex network of postsynaptic and plasticity-controlling proteins, including PSD95, a major constituent of the postsynaptic density that acts as a bridge between Glutamate receptors, calcium/calmodulin-dependent protein kinase, and scaffolding proteins such as Dlgap1 and Shank proteins. The module also contains a submodule of dendritic Ca^++^ channels, and a module containing proteins that regulate the spine cytoskeleton, such as Baiap2 [78], and regulators of local translation such as Cyfip1 [79]. A second related module shows as hub β-catenin (Ctnnb1), and is subdivided in a module delated to junctions containing, for example, Vinculin (Vcl), which can promote growth of neuronal processes [80]; the other submodule contains as hub the kinase Gsk3, a kinase well known to play a central role in plasticity [81]. Importantly, while the increased levels of this module specifically identify the postsynaptic component of glutamatergic synapses, we did not find any increase in postsynaptic GABAergic synapses, indicating an imbalance between excitation and inhibition. 

The postsynaptic module is directly connected, via CamK2b, to a module that has, as hub, the CASK kinase, known to interact with CamKII to regulate neuronal growth and plasticity [54]. While this module specifically identifies the postsynaptic component of glutamatergic synapses, we did not find any increase in postsynaptic GABAergic synapses, indicating an imbalance between excitation and inhibition. 

The network of postsynaptic and plasticity-controlling proteins was mirrored by a cluster of increased levels in presynaptic proteins, including Snap25, Rims1, Basson, Piccolo, and Syntaxins.

The plasticizing effect of physical activity was further confirmed by its impact on other modules directly connected to plasticity, including proteostasis (Hsp90ab1), 19S Proteasome, the clathrin/adaptor protein complex 2 (AP-2), and proteins involved in either microtubule dynamics (Ankryns with hubs Ank3), or in the regulation of the mobility of presynaptic vescicles (Rab6a). Moreover, there were reduced levels of proteins known to act as brakes for plasticity, such as Tnc, Acan, and Hapln1, which are components of the perineuronal nets [50,51], and the myelin oligodendrocyte protein Mog [82]. 

Most importantly, the OD shift in response to MD promoted by running was damped by diet supplementation with the antioxidants vit E and C, even if the C/I VEP ratio in animals subjected to the combined treatment (running + vitamins) was slightly lower than that in sedentary animals fed with the standard dietary regimen. This may possibly derive from a redundancy in the mechanisms through which physical exercise induces plasticity, some of which may be directly triggered in the cortex by the kinesthetic feedback [35,72,83].

Importantly, vitamin treatment induced a down-regulation of proteins involved in visual cortex plasticity in exercised animals, as shown by the significant case of the proteins belonging to the pathway “glutamatergic synapse”, without any effect on GABAergic synapses. 

Treatment with antioxidants also induced significant protein changes in the sedentary condition. In particular, it induced a marked down-regulation of the oxidative phosphorylation pathway, opening the possibility that a direct action of the vitamins on the visual cortex could be responsible for dampening the positive effects of physical activity effects on plasticity. However, wheel running significantly down-regulated the expression of Catalase, Sod1, and of the Glutathione metabolism pathway, suggesting that a local increase of oxidative stress is unlikely. 

A related possibility is that the induction of plasticity in exercised animals relies on an increased activity of the respiratory chain, as suggested by the observation that synaptic mitochondria fuel local translation during plasticity [84]. However, since treatment with the antioxidants did not block plasticity during the CP, this is a strong indication that the effects of vitamins are actually not direct on the cortex, or else that the plasticity mechanisms set in motion by exercise are different from those underlying developmentally regulated plasticity. 

**Figure 7:**
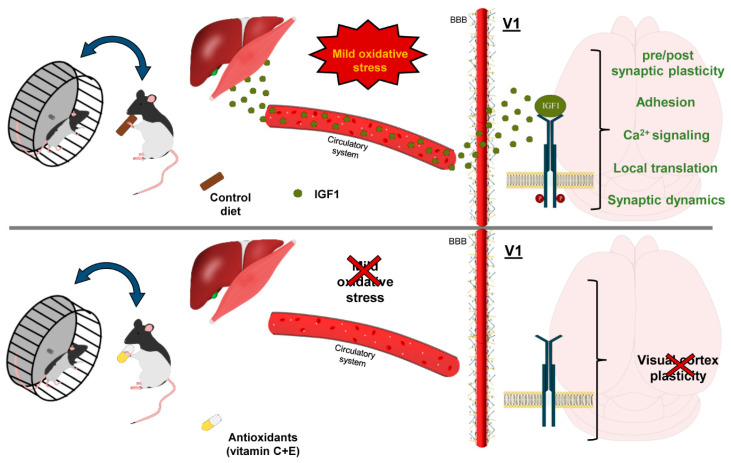
A schematic model of mithormesis in response to physical activity and of the antioxidant effects.

In view of these findings, we propose a model in which physical activity stimulates mitochondrial oxidative metabolism in skeletal muscles, and, in parallel, leads to IGF-1 release from the muscles themselves [85] and/or from the liver [86] (Figure 7). Circulating IGF-1 enters the brain through the blood brain barrier [47], activating its specific receptors in the visual cortex and leading to stimulation of intracellular pathways involved in visual cortex plasticity [87,88,89,90]. This may reopen susceptibility to the effects of MD beyond the closure of the CP. Supplementation with high doses of the antioxidants vitamins E and C limits IGF-1 release [91] in running subjects, an effect possibly due to a decreased activity of the COX-2 enzyme [92]. The resulting decrease of circulating IGF-1 may eventually dampen the plasticizing effects set in motion by physical exercise (Figure 7).

## 5. Conclusions

These results unify the local and systemic effects of physical exercise under the mitohormesis theory and underline the importance of mitochondria in the induction of plasticity. 

## Figures and Tables

**Table 1:** Statistical analysis summary.

Experiment	Groups and Numerosity	Normality Test (Shapiro Test)	Equal Variance Test	Comparison	Multiple Comparisons Correction
Electrophysiology (adults)	RC = 9; SC = 7; RV = 9; SV = 7; Naïve + vit = 5; Naïve + ctrl = 5	Passed, *p* = 0.195	Passed, *p* = 0.288	Two Way ANOVA	Holm–Sidak
Electrophysiology (juveniles)	SV = 6; SC n = 5	Passed, *p* = 0.332	Passed *p* = 0.522	Two-tailed T-test	N.A.
Β-hydroxybutyrate assay	RC = 6; SC = 6; SV = 6; RV = 6	Passed, *p* = 0.9	Passed, *p* = 0.933	Two Way ANOVA	Holm–Sidak
IGF-1 assay	RC = 8; SC = 7; RV = 8; SV = 7	Passed, *p* = 0.834	Passed, *p* = 0.131	Two Way ANOVA	Holm–Sidak
IGF-1 phosphorylated receptor (WB)	RC = 6; SC = 6; RV = 6; SV = 6	Passed, *p* = 0.422	Passed, *p* = 0.889	Two Way ANOVA	Holm–Sidak
Distance covered while running	RV = 9 RC = 9	Failed, *p* < 0.05	Passed *p* = 0.438	Two Way RM ANOVA on ranks	N.A.

## Data Availability

The dataset deriving from proteomic data are presented in Tables S1 and S2. Other datasets generated in the present study are available from the corresponding author on reasonable request.

## Supplementary Materials

The following supporting information can be downloaded at: https://www.mdpi.com/article/10.3390/cells12010048/s1, Table S1: candidate list, Table S2: list of significantly-affected proteins.
